# High expression of galectin-7 associates with poor overall survival in patients with non-metastatic clear-cell renal cell carcinoma

**DOI:** 10.18632/oncotarget.9749

**Published:** 2016-05-31

**Authors:** Jieti Wang, Yidong Liu, Yuanfeng Yang, Zhiying Xu, Guodong Zhang, Zheng Liu, Hangcheng Fu, Zewei Wang, Haiou Liu, Jiejie Xu

**Affiliations:** ^1^ Department of Biochemistry and Molecular Biology, School of Basic Medical Sciences, Fudan University, Shanghai 200032, China; ^2^ Department of Urology, Zhongshan Hospital, Fudan University, Shanghai 200032, China; ^3^ Shanghai Key Laboratory of Female Reproductive Endocrine Related Diseases, Hospital of Obstetrics and Gynecology, Fudan University, Shanghai 200011, China

**Keywords:** clear-cell renal cell carcinoma, galectin-7, prognosis, nomogram

## Abstract

**Background:**

Galectin-7, has a controversial role in tumor progression, can either suppress tumor growth or induce chemoresistance depends on different tumor histology types. The aim was to appraise Galectin-7 expression on the overall survival (OS) of patients with non-metastatic clear cell renal cell carcinoma (ccRCC) following surgery.

**Results:**

High galectin-7 expression was specifically correlated with necrosis (*P* = 0.015). Multivariate analysis confirmed galectin-7 as an independent prognosticator for OS (*P* = 0.005). High galectin-7 expression suggested poor OS (*P* < 0.001), particularly with UISS intermediate and high score groups. Notably, the predictive accuracy of the traditional prognostic scores was improved when combined with galectin-7 expression.

**Materials and Methods:**

We retrospectively enrolled 416 patients who underwent nephrectomy at a single institute between 2008 and 2009 and detected their intratumor galectin-7 expression by immunohistochemistry. Kaplan-Meier method was conducted to plot survival curves and multivariate cox regression analysis for potential independent prognostic factors on OS. A nomogram was constructed with concordance index (C-index) and Akaike's Information Criteria (AIC) to appraise prognostic accuracy of different models.

**Conclusions:**

High galectin-7 expression is an independent adverse predictor for survival. Evaluation of galectin-7 could help guide postsurgical management for non-metastatic ccRCC patients.

## INTRODUCTION

Renal cell carcinoma, the most prevalent solid tumor of kidney malignancies, represents 2–3% of all human cancers [1]. Clear cell renal cell carcinoma, the commonest histological type of RCCs, has the worst prognosis with an average 5-year survival at 71% [2]. Although the prognostic values of traditional clinic-pathologic factors, such as pathological T, N stages, metastasis, the presence of necrosis, sarcomatoid and LVI, as well as ECOG-PS have been addressed, their combined impacts on ccRCC prognosis is however scattered [3]. Recently, novel models combining conventional clinic-pathologic factors with molecular biomarkers had been established to provide more precise prediction for overall and recurrence-free survival, aid patient management and treatment [4–6]. Hence, it's promising to seek new molecules to optimize present prognostic systems.

Galectins, a subgroup of the lectin family, known for their ability to bind to N-linked or O-linked glycosylated β-galactoside sugars, universally participate in cancer initiation, progression and metastasis [7]. It's reported that galectins expression changes in carcinoma cells under micro-environmental stress conditions during tumor development [8], thereby can be potentially novel prognosticators and targets [9]. Galectin-7, a homodimer galectin, is implicated in various biological effects including cell proliferation, differentiation, apoptosis, collective cell migration and immune-modulatory [10–13], which plays a completely discrepant role in different types of cancers. On the one hand, galectin-7 was affirmed as a p53 induced protein and may participate in the pro-apoptotic function of p53 [14], thereby suppresses tumor growth [15]. Ueda, S et al. verified it as a tumor suppressor in gastric cancer [16] and Tsai, CJ et al reported elevated galectin-7 expression as a benign predictor in squamous cell carcinoma of the cervix and was associated with better outcomes after radiotherapy [17]. On the other hand, galectin-7 can boost MMP-9 expression and impede p53 expression, thereby promotes tumorigeneses and induces chemoresistance [18–20]. Demers, M. et al. and Labrie, M. et al. reported galectin-7 was associated with cancer metastasis and chemo-resistant in breast and ovarian cancers, respectively [21, 22]. Although all these important roles of galectin-7 in cancers of different histologic types had been identified, there haven't any reports about the relationship between galetin-7 expression and RCC, whether it can promote or suppress tumor progression in RCC remained uncertain and its prognostic power in RCC needed to be declared.

In this study, we evaluated the galectin-7 expression in tumor tissues by immunohistochemistry and analyzed the relationship between galectin-7 expression and overall survival in 416 patients with non-metastasis ccRCC. Furthermore, a nomogram was established to help predict prognosis and guide management for ccRCC patients without metastatic diseases.

## RESULTS

### Galectin-7 expression and clinical characteristics in patients with non-metastasis ccRCC

**Figure 1:**
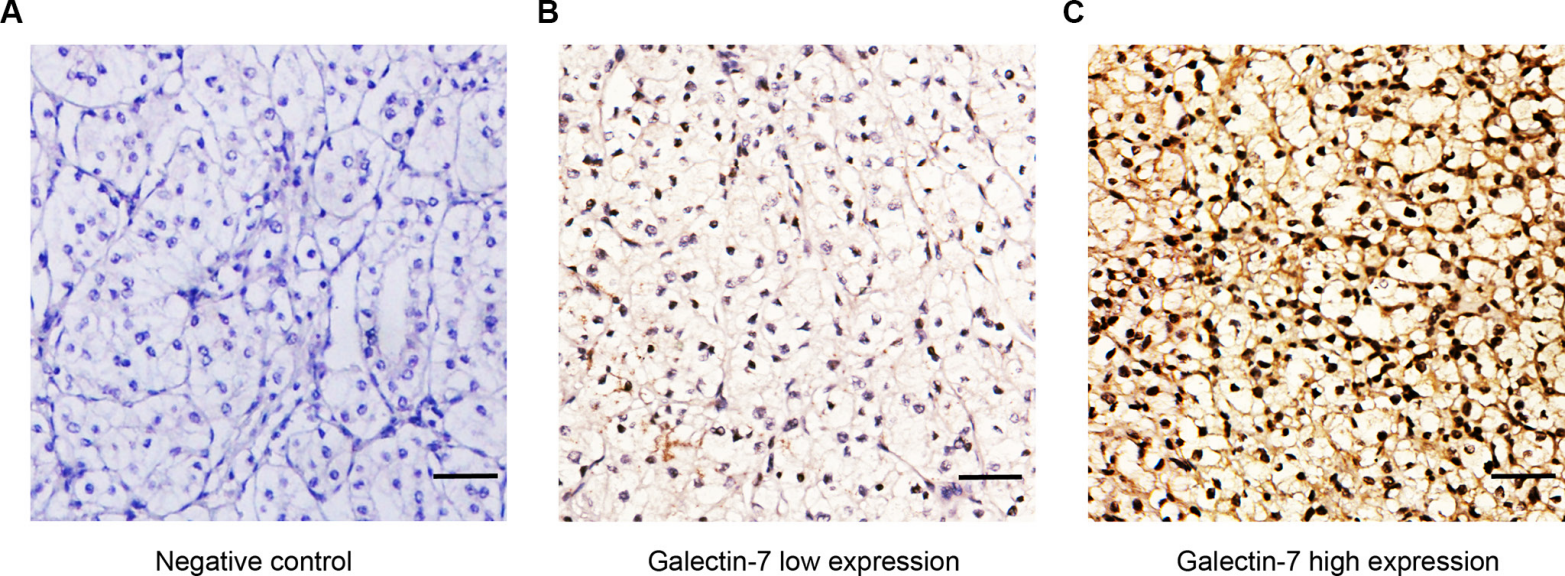
Representative immunohistochemistry staining pictures of ccRCC tissue sections in galectin-7 expression (**A**) Negative control. (**B**) Intratumor low galectin-7 expression. (**C**) Intratumor high galectin-7 expression. Scale bar: 50 μm (original magnification × 200).

After applying initial exclusion criteria, the expressions of galectin-7 were assessed in an aggregate of 416 non-metastatic ccRCC samples by immunohistochemistry staining analysis. As the pictures presented, the expression of galectin-7 dominantly presented in cytoplasm and nuclear of tumor cells and the expression level varied in patient specimens (Figure 1). The H-score of galectin-7 ranged from 0 to 208, and according to the “minimum *P* value” measure conducted by X-tile, 416 patients were divided into galectin-7 low group (score, 0–80; *n* = 255) and galectin-7 high group (score, 81–208; *n* = 161).

According to the correlation analyses, higher galectin-7 expression was associated with the presence of necrosis (*P* = 0.015), while other clinic-pathologic variables were presented to have no significant correlation with galectin-7. Furthermore, there was no significant discrepancy between the patients in galectin-7 high and low group regarding UISS score, Leibovich score and SSIGN score (Table 1).

**Table 1:** Correlations between Galectin-7 expression and clinical characteristics in non-metastasis ccRCC patients

Characteristic	Patients (*n* = 416)		Galectin7 expression		*P*
	No.	%	Low (*n*= 255)	High (*n* = 161)	
Age at surgery, years					0.790
Mean ± SD	55.37 ± 12.15		54.47 ± 12.20	56.79 ± 11.96	
Gender					0.272
Male	296	71.2	176	120	
Female	120	28.8	79	41	
Tumor size, cm					0.098
Mean ± SD	4.23 ± 2.36		4.18 ± 2.25	4.31 ± 2.52	
Pathological T stage					0.546
T1	294	70.7	185	109	
T2	25	6.0	15	10	
T3	97	23.3	55	42	
Necrosis					**0.015**
Absent	336	80.8	216	120	
Present	80	19.2	39	41	
Sarcomatoid*					0.438
Absent	409	98.3	252	257	
Present	7	1.7	3	4	
LVI					0.066
Absent	316	76.0	202	114	
Present	100	24.0	53	47	
Fuhrman grade					0.067
1	78	18.8	58	20	
2	196	47.1	112	84	
3	95	22.8	57	38	
4	47	11.3	28	19	
ECOG-PS					0.051
0	348	83.7	221	127	
≥ 1	68	16.3	34	34	
UISS score					0.434
LR	191	45.9	122	69	
IR	198	47.6	119	79	
HR	27	6.5	14	13	
Leibovich score					0.230
LR	231	55.5	149	82	
IR	148	35.6	87	61	
HR	37	8.9	19	18	
SSIGN score					0.079
LR	307	73.8	198	109	
IR	101	24.3	53	48	
HR	8	1.9	4	4	

LVI = lymphovascular invasion; ECOG PS = Eastern Cooperative Oncology Group performance status; UISS = UCLA Integrated Staging System; SSIGN = Mayo clinic stage, size, grade, and necrosis; LR = low risk; IR = intermediate risk; HR = high risk.

* Fisher's exact test; chi-square test for all the other categorical variables and students'*t*-test for all continuous variables.

### High expression of galectin-7 is associated with worse overall survival

**Figure 2:**
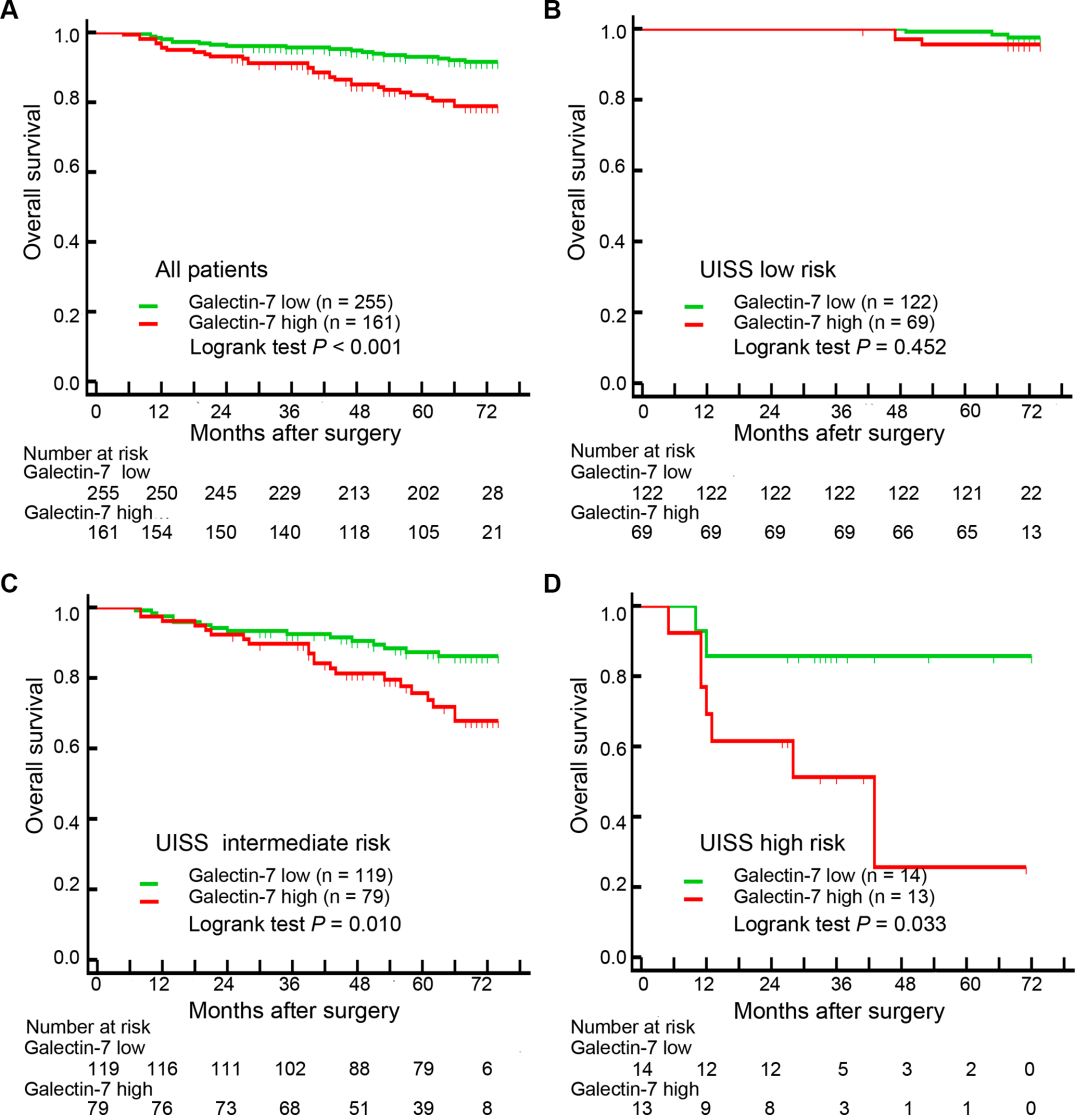
Prognostic power of galectin-7 in non-metastasis ccRCC patients (**A**) Kaplan-Meier curves of OS based on intratumor galectin-7 expression levels. (**B**–**D**) Kaplan-Meier curves of OS based on intratumor galectin-7 expression levels in UISS low risk group, UISS intermediate risk group and UISS high risk group.

The median follow-up was 70 months (range, 42–76 months) and 51 (12.3%) patients died during the period. Kaplan-Meier survival analysis revealed that galectin-7 was significantly associated with OS (high vs low; hazard ratio, 95% CI, 2.662, 1.521–4.658; Log-rank test *P* < 0.001) in non-metastasis ccRCC patients and the 5-year overall survival probability in galectin-7 low group is 93.0% while galectin-7 high group has an overall survival probability of 82.1% (Figure 2A).

To further confirm the findings, we divided 416 patients into 3 risk groups according to the UISS score: low risk (score 1; *n* = 191, 45.9%), intermediate risk (score 2; *n* = 198, 47.6%) and high risk (score 3; *n* = 27, 6.5%). Kaplan-Meier survival analyses presented that the remarkable difference between galectin-7 high and low patients was dominantly lay in UISS intermediate and high risk groups (Log-rank *P* = 0.010 and 0.033 respectively; Figure 1B–1D).

### Galectin-7 expression as an independent prognosticator in non-metastasis ccRCC patients

**Figure 3:**
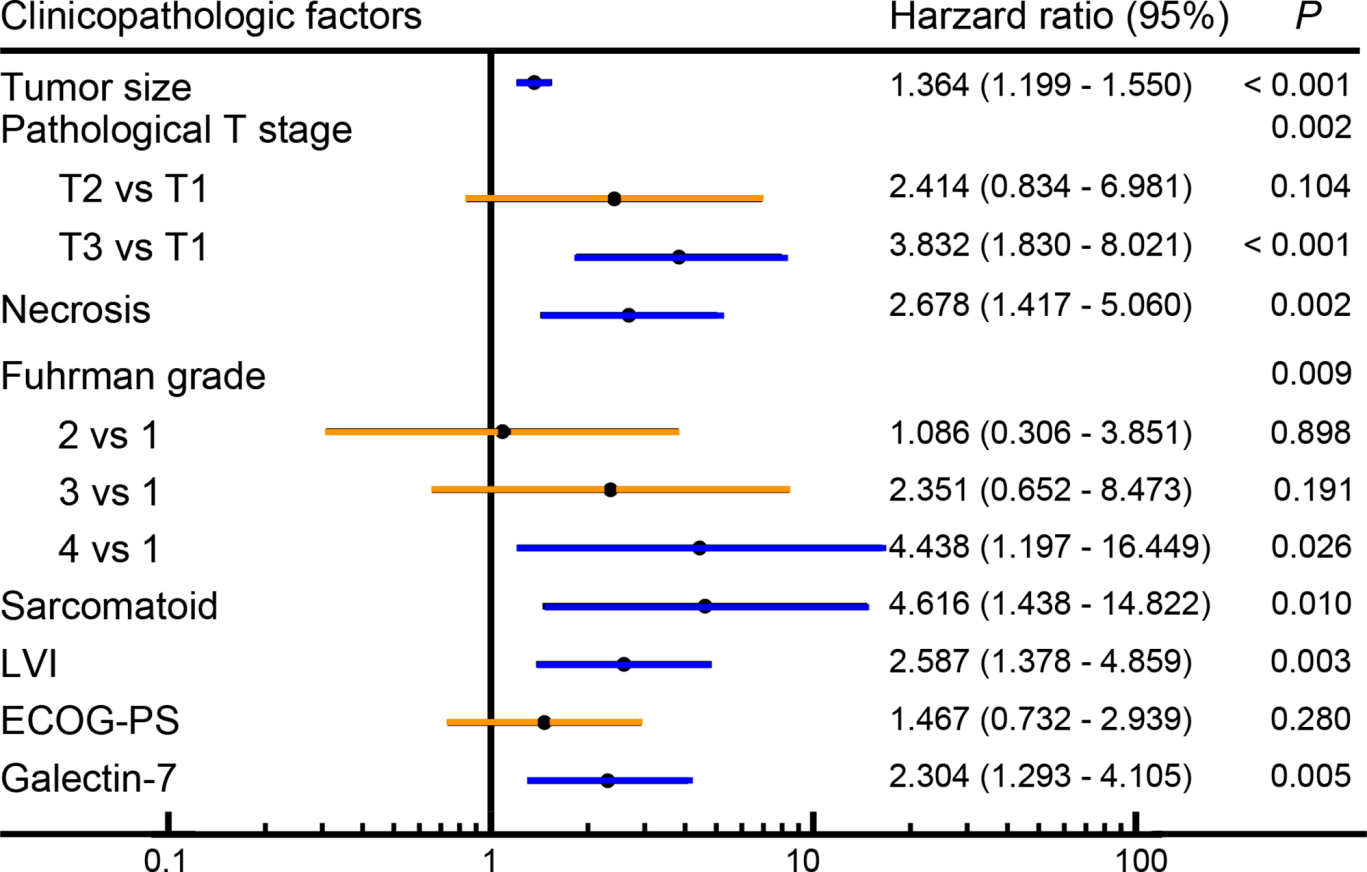
Multivariate Cox regression analysis of clinic-pathologic factors for overall survival Forest plot presented results of multivariate Cox regression analysis of all available prognostic factors (tumor size, pathological T stage, necrosis, sarcomatoid, LVI, ECOG-PS and galectin-7) in patients with non-metastasis ccRCC.

We conducted multivariate Cox regression analysis to apprise the independent prognostic power of galectin-7 and all accessible clinic-pathologic variables (tumor size, pathological T-stage, necrosis, Fuhrman grade, sarcomatoid, LVI and ECOG-PS) in non-metastasis ccRCC. Results indicated that tumor size (*P* < 0.001), pathological T stage (*P* = 0.002), necrosis (*P* = 0.002), Fuhrman grade (*P* = 0.009), sarcomatoid (*P* = 0.010), LVI (*P* = 0.003) and galectin-7 (*P* = 0.003) were independently predictive factors of OS, while ECOG-PS (*P* = 0.280) showed no significance (Figure 3). Furthermore, multivariate Cox regression analysis were conducted in UISS subgroups. Considering the wide variation on UISS high subgroup, we combined UISS intermediate and high subgroups to UISS higher risk subgroup. As the results presented, tumor size, pathological T stage, necrosis, sarcomatoid, LVI and galectin-7 were independently predictive factors of OS, while Fuhrman grade and showed no significance.(Supplementary Figure S1).

Furthermore, we investigated whether the galectin-7 expression signature can help improve the predictive accuracy of known prognostic models (UISS score, Leibovich score and SSIGN score). As the results presented, cooperating galectin-7 expression signature with these models manifests a larger C-index (0.743 vs 0.779, 0.816 vs 0.829, 0.805 vs 0.822, respectively) and a smaller AIC (556.8 vs 547.7, 512.4 vs 504.1, 512.8 vs 506.2, respectively) than the original ones and have better predictive accuracy (Table 2).

**Table 2:** Comparison of the predictive accuracy of the established nomogram with known prognostic models

Prognostic models	C-index*	AIC#
galectin-7	0.619	591.0
UISS score	0.743	556.8
Leibovich score	0.816	512.4
SSIGN score	0.805	512.8
UISS + galectin-7	0.779	547.7
Leibovich + galectin-7	0.829	504.1
SSIGN + galectin-7	0.822	506.2
Nomogram	0.854	474.5

* A larger C-index suggests a better discriminatory power.

# A smaller AIC suggests a better discriminatory power.

### Establishment and validation of prognostic nomogram for OS

**Figure 4:**
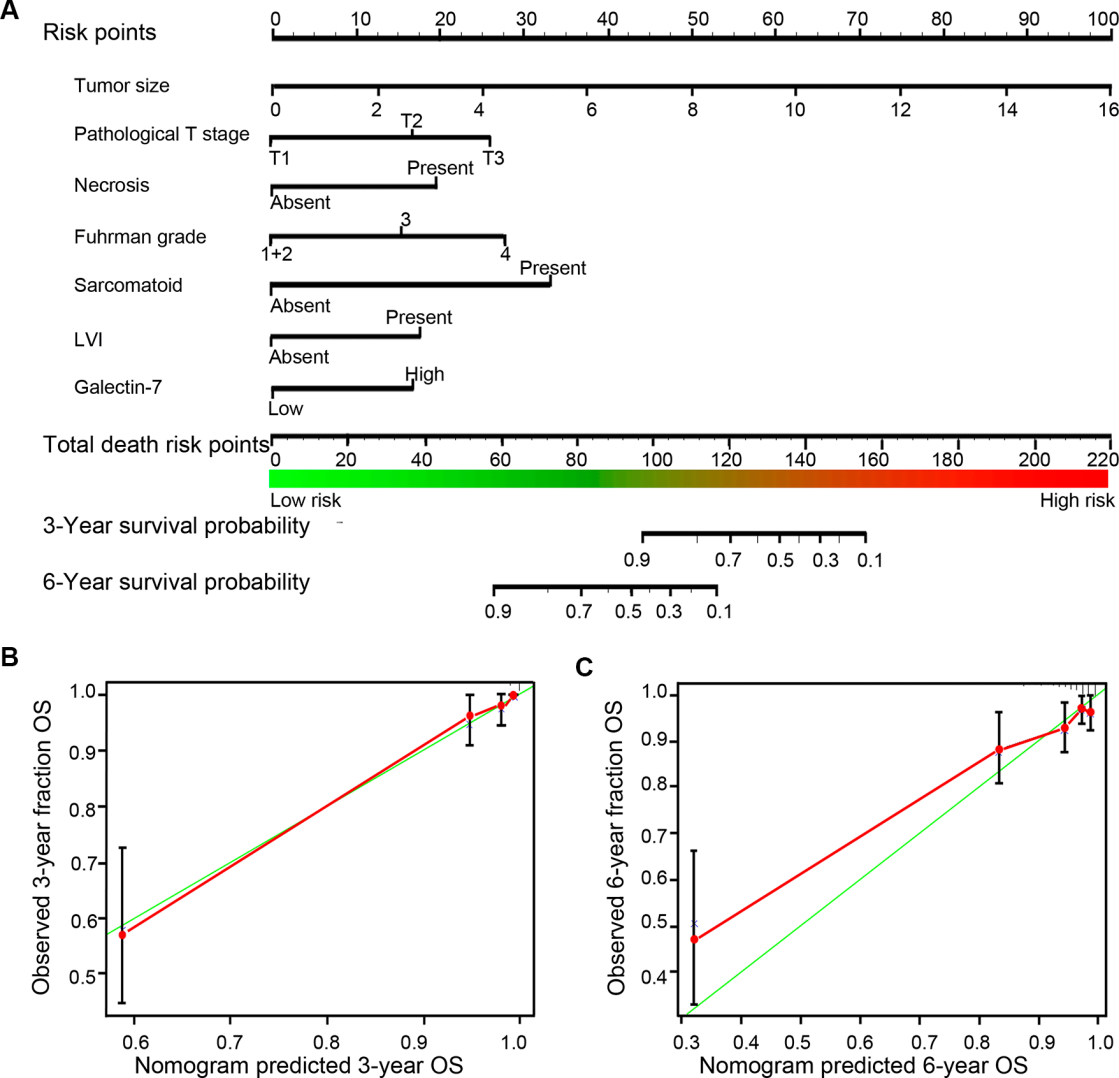
Nomogram and calibration plots for prognostic prediction of postoperative non-metastasis ccRCC patients (**A**) 6 independent prognostic factors (Size, pathological T stage, necrosis, Fuhrman grade, sarcomatoid and galectin-7) were used to establish the nomogram, predicting death risk of 3-year and 6-year OS. (**B**–**C**) Calibration curves for predicting 3-year and 6-year OS of non-metastasis ccRCC patients.

According to preceding analyses, Fuhrman grade 2 can't well different from Fuhrman grade 1 (hazard ratio = 1.080; 95% CI, 0.306–3.851; *P* = 0.898) for predicting OS (Figure 3), hence we combined Fuhrman grade 1 and 2 to single Fuhrman 1 + 2 for further nomogram building-up without compromising the model robustness. All the independent predictive factors were cooperated to establish a prognostic nomogram (Figure 4A). The nomogram illustrated that tumor size contributed largest to the prognostication (weighted ratio = 0.414), while necrosis, LVI as well as galectin-7 showed a minor impact on outcome (weight ratio = 0.079, 0.075, 0.071, respectively). The calibration plots manifested an excellent consistency in bootstrap analysis between the calculated and real observation for 3-year and 6-year OS (Figure 4B–4C).

The comparison among the established nomogram and known predictive models for OS was conducted to assess the predictive power of the new model. It's displayed that the Harrell's C-index for the built-up nomogram to predict OS (0.854) was significantly larger than that of the UISS score (0.743), Leibovich score (0.816) and SSIGN score (0.805), while the AIC was smaller than these models (474.5 vs 556.8, 512.4 and 512.8, respectively) (Table 2). This superior performance of nomogram manifested it's an excellent predictive models for OS in patients with non-metastasis ccRCC.

## DISCUSSION

Our study first reported the independent prognostic power of galectin-7 for OS in non-metastasis ccRCC patients. Moreover, galectin-7 manifests a better discriminative power in UISS intermediate and high risk subgroups, which can help optimize the traditional score system in guiding management of patients with higher UISS score. The nomogram integrating tumor size, pathological T stage, necrosis, Fuhrman grade, sarcomatoid, LVI and galectin-7 expression shows a better prognostic accuracy in OS than the traditional models, thus has a better effectiveness in non-metastasis ccRCC patients OS prediction.

Consistence with reports in breast, ovarian and oral squamous cell carcinoma [19, 22, 23], elevated galectin-7 expression acted as a reverse predictive factor in non-metastasis ccRCC patients. This finding may result from the function of galectin-7 in enhancing tumor cells aggressiveness and inducing chemo-resistance [19]. However, our study excluded these patients with metastasis ccRCC, yet the expression level of galectin-7 still performed well in predicting overall survival, which revealed galectin-7 may participate in other cellular biochemistry progresses in ccRCC progression and metastasis beyond the known functions. Furthermore, previous study revealed that in normal kidney tissues, galectin-7 can selectively bind to cilia, increase cilia length and promote polarized epithelial repairing [24]. However, in cancerous renal tissue, galectin-7 may manifest completely different functions. Take the uterus for examples, galectin-7 up-regulate MMP-9 expression, thereby promotes tumor progression and metastasis in cervical squamous carcinoma [25], while in normal uterus, it promotes uterine repair following menstruation [26]. Our study revealed the correlation of galectin-7 expression and overall survival in non-metastasis ccRCC, which shed a light on the potentially different functions of galectin-7 in ccRCC from normal kidney tissues.

Similar to galectin-1, 3, 8 and 9, high galectin-7 expression acts as an adverse predictor for survival in patients with RCC [27–30]. In RCC, galetin-1 can activate HIF-1α-mTOR signaling axis, enhance the migration ability of tumor cells, and promote tumor progression [27]. Elevated galectin-3 expression was correlated with higher Fuhrman grade in RCC and predict worse overall survival [28]. Galectin-9 expression was positively associated with tumor size, Fuhrman grade, and necrosis and was an independent prognostic indicator for OS in ccRCC [30]. However, different from galectin-1,3 and 9, high galetin-7 expression is specifically relevant to the present of necrosis in non-metastasis ccRCC, which is similar to the characteristic of galectin-8 in pT1 ccRCC [29]. This specific relevance might derived from the ability of galectin-7 in enhancing the pro-apoptosis function of p53 and triggering further cell death signaling pathways. Considering the possible function of galectin-7 expression in activating cell necrosis in ccRCC, it may be targeted to decrease necrosis, lower inflammatory burden [31] in ccRCC and improve patients survival.

Despite the clinical significance of galectin-7 in non-metastasis ccRCC has been presented, several constraints of this study require further discussion. First, given the heterogeneous feature of ccRCC and the single hospital sampled population of our study, our result might overestimate the prognostic power of galectin-7. Second, the relative expression level of galectin-7 was cutoff based on the intensity of immunohistochemistry staining, which made the intermediate intensity hard to define. The more quantitative measurement, such as serum test might be more dependable. Third, our study constrain the population in non-metastasis ccRCC, the potential association between galectin-7 and metastasis ccRCC or other RCC needs to be explored.

In conclusion, our study galectin-7 expression in tumor tissue as a potential independent predictive factor for OS in patients with non-metastasis ccRCC. Integrating galectin-7 expression with conventional clinical pathologic factor could establish a novel nomogram which shows a better prognostic accuracy than traditional score systems and helps guiding patients management.

## MATERIALS AND METHODS

### Patients

The database included 416 consecutive patients with non-metastasis ccRCC at Zhongshan Hospital (Shanghai, China) between 2008 and 2009. The original inclusive criteria: (a) confirmed post-operative histopathology diagnosis as clear cell RCC; (b) no radiotherapy or chemotherapy before surgery; (c) with *N* = 0, *M* = 0 and T ≠ 4 based on 2010 AJCC TNM classification [32]. This study was admitted by the hospital's ethics review committee, and informed consents were accessed by phone-call from each patient. The information of clinical characteristics were collected for each patients and the UISS, Leibovich and SSIGN scores were collected according to original scoring protocols [33–35], respectively. Based on these predictive models, all patients were divided into 3 groups: low risk group (LR; UISS = 1, Leibovich = 0–2, SSIGN = 0–2, respectively), intermediate risk group (IR; UISS = 2; Leibovich = 3–5, SSIGN = 3–5, respectively) and high risk group (IR; UISS = 3; Leibovich ≥ 6, SSIGN ≥ 6, respectively).

### Tissue microarray and immunohistochemistry

Primary formalin-fixed, paraffin-embedded tissues were collected from Zhongshan Hospital (Shanghai, China) and tissue microarrays and immunohistochemistry were performed as previously described [36]. As briefly described, all samples were reviewed histologically by hematoxylin and eosinstaining, and representative areas were marked on the paraffin blocks away from necrotic and hemorrhagic materials. Duplicate 1.0-mmtissuecores from 2 different areas were used to construct the tissue microarray. Sections from the TMA blocks were cut at 4 um. Anti-galectin-7 antibody (1:100, Abcam, ab108623) was used to perform the immunohistochemistry staining. Immunohistochemistry sections were scanned by an automated microscopy system (Leica DM6000 B, Leica Microsystems GmbH, Mannheim, Germany), images were captured by Leica CV-M2CL camera and sent to 2 pathologists without knowing the patient outcomes to score (H-score) as previously described [36].

### Statistical analysis

X-tile analysis was performed to dichotomize all 416 patients into galectin-7 low and high groups based on the intensity of immunohistochemistry straining. Fisher's exact test, chi-square test and independent samples *t*-test were conducted to appraise the correlation of galectin-7 expression and clinic-pathologic factors as appropriate. Kaplan-Meier method was used to plot survival curves. Multivariate Cox regression analyses was conducted to explore independent predictive factors. The concordance index (C-index) and Akaike's Information Criteria (AIC) were used to evaluate the predictive power of different models [37]. Statistical analyses were conducted with X-tile 3.6.1 (Yale University, New Haven), MedCalc 11.4.2.0 (MedCalc, Mariakerke), IBM SPSS Statistics v21.0 (IBM Corp, Armonk), R 3.2.3 (R Foundation for Statistical Computing, Vienna) and Stata SE 12.0 (Stata, College Station, TX).

## SUPPLEMENTARY FIGURES


