# Supplementary material for: High expression of galectin-7 associates with poor overall survival in patients with non-metastatic clear-cell renal cell carcinoma
Source: Oncotarget. 2016 May 31;7(27):41986–95. doi: 10.18632/oncotarget.9749 (PMC5173110; doi:10.18632/oncotarget.9749)
Supplement: Supplementary file 1 [file oncotarget-07-41986-s001.pdf]

High expression of galectin-7 associates with poor overall survival in patients with non-metastatic clear-cell renal cell carcinoma

Supplementary Materials

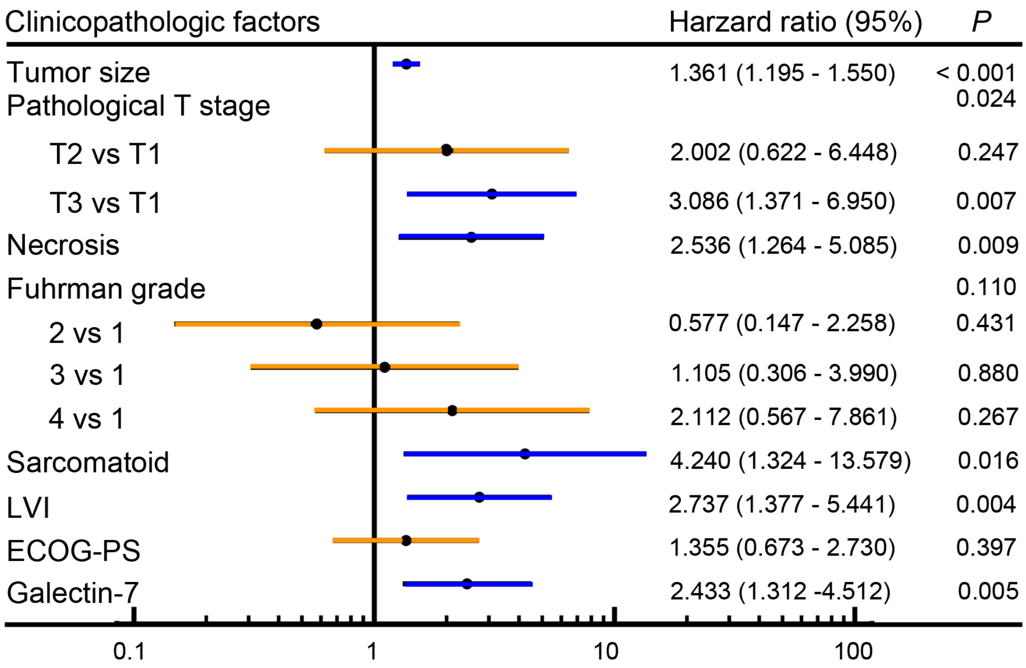

Supplementary Figure S1: Multivariate Cox regression analysis of clinic-pathologic factors for overall survival in UISS higher risk subgroup. Forest plot presented results of multivariate Cox regression analysis of prognostic factors in UISS higher (intermediate and high) risk subgroup.
